# The Effects of Lutein and Zeaxanthin Supplementation on Cognitive Function in Adults With Self-Reported Mild Cognitive Complaints: A Randomized, Double-Blind, Placebo-Controlled Study

**DOI:** 10.3389/fnut.2022.843512

**Published:** 2022-02-17

**Authors:** Adrian L. Lopresti, Stephen J. Smith, Peter D. Drummond

**Affiliations:** ^1^Clinical Research Australia, Perth, WA, Australia; ^2^Healthy Ageing Research Centre and Discipline of Psychology, College of Science, Health, Engineering and Education, Murdoch University, Perth, WA, Australia

**Keywords:** lutein, zeaxanthin, carotenoids, mild cognitive impairment, memory, randomized clinical trial

## Abstract

**Background:**

Lutein and zeaxanthin are fat-soluble, dietary carotenoids with high concentrations in human brain tissue. There have been a number studies confirming an association between lutein and zeaxanthin and cognitive function.

**Purpose:**

Examine the effects of lutein and zeaxanthin supplementation on cognitive function in adults with self-reported cognitive complaints.

**Study Design:**

Two-arm, parallel-group, 6-month, randomized, double-blind, placebo-controlled trial.

**Methods:**

Ninety volunteers aged 40–75 years received either 10 mg of lutein and 2 mg of zeaxanthin, once daily or a placebo. Outcome measures included computer-based cognitive tasks, the Cognitive Failures Questionnaire, Behavior Rating Inventory of Executive Function, Profile of Mood States, and the Patient-Reported Outcomes Measurement Information System-29.

**Results:**

Compared to the placebo, lutein and zeaxanthin supplementation was associated with greater improvements in visual episodic memory (*p* = 0.005) and visual learning (*p* = 0.001). However, there were no other statistically-significant differences in performance on the other assessed cognitive tests or self-report questionnaires. Lutein and zeaxanthin supplementation was well-tolerated with no reports of significant adverse effects.

**Conclusion:**

The results from this trial suggest that 6-months of supplementation with lutein and zeaxanthin may improve visual memory and learning in community-dwelling adults with self-reported cognitive complaints. However, it had no other effect on other computer-based measures of cognitive performance or self-report measures of cognition, memory, mood, or physical function.

## Introduction

Lutein and zeaxanthin are fat-soluble nutrients forming part of the carotenoid family. Lutein is found in dark green leafy vegetables such as kale and spinach and in egg yolks and corn (1). Zeaxanthin is more prominently found in yellow and orange foods such as egg yolks, corn, orange capsicums, tangerines, persimmons, mandarins, and oranges (2, 3). In the body, lutein and zeaxanthin are found in eye, brain, breast and adipose tissue. Although lutein is not the major carotenoid in our diet, it is the carotenoid of the highest concentration in human brain tissue (4, 5). In fact, lutein and zeaxanthin account for 66 to 77% of the total carotenoid concentration in human brain tissue (6). Lutein and zeaxanthin have been identified in the hippocampus, cerebellum, and frontal, occipital, and temporal cortices (5, 7–9); and due to their powerful antioxidant and anti-inflammatory properties (10, 11), interest in their neuroprotective effects is increasing.

Studies examining the relationship between the dietary intake of carotenoids, including lutein and zeaxanthin, have demonstrated a generally positive relationship between lutein and zeaxanthin intake and cognitive health. For example, a higher dietary intake of lutein and/or zeaxanthin was associated with a lower risk of experiencing moderate-to-poor cognitive function in middle-aged women (12), better immediate and delayed word recall in older adults (13), and higher scores on several cognitive-based measures in adults over the age of 60 years (14). Moreover, higher plasma concentrations of lutein and/or zeaxanthin were associated with better cognitive function in older adults (15, 16), visual-spatial functioning in older adults (17), and relational memory performance in young and middle-aged adults (18). Macular pigment optical density (MPOD), which provide a measure of lutein and zeaxanthin concentration in the brain (8, 19), was also associated with better cognitive performance in older-age adults (16), adults with mild cognitive impairment (20), and in adults with age-related macular degeneration (21). However, despite these findings, results from randomized controlled trials have been inconsistent. For example, 12 months of supplementation with 10 mg of lutein and 2 mg of zeaxanthin were associated with improvements in complex attention and cognitive flexibility in community-dwelling older adults (22), buffered cognitive decline on a verbal learning task in older adults (22), and increased spatial memory in young, healthy adults (23). In this latter study, participants who experienced increases in MPOD, irrespective of group allocation, experienced improvements in visual memory, complex attention, and reasoning ability. However, in a large 5-year study on older-age adults with intermediate or advanced age-related macular degeneration, lutein and zeaxanthin supplementation did not change cognitive function as measured by several telephone-administered cognitive tasks (24). These inconsistent findings are likely due to differences in the population recruited, outcome measures used, and treatment duration. Exposure to dietary sources of lutein and zeaxanthin are also likely to confound results as it is challenging to conduct clinical trials on nutrients that are found in everyday foods and may be consumed by participants daily. The finding by Renzi-Hammond et al. (23) where improvements in cognitive performance occurred in participants who experienced increases in MPOD, irrespective of group allocation, suggests increases in brain concentration of lutein and zeaxanthin (either via dietary sources or supplementation) are necessary for improvements in cognition to be realized.

The aims of this trial were to add to the existing body of research and to examine the effects and tolerability of lutein and zeaxanthin supplementation over 6 months on cognitive performance in community-dwelling adults with self-reported cognitive complaints. In contrast to most previous studies, supplementation was for 6 months, as opposed to primarily 12-month trials, and in a middle-to-older age cohort with self-reported cognitive complaints. It was hypothesized that supplementation with lutein and zeaxanthin would improve cognitive performance as measured by computer-based tasks and self-report measures.

## Materials and Methods

### Study Design

**Figure 1:**
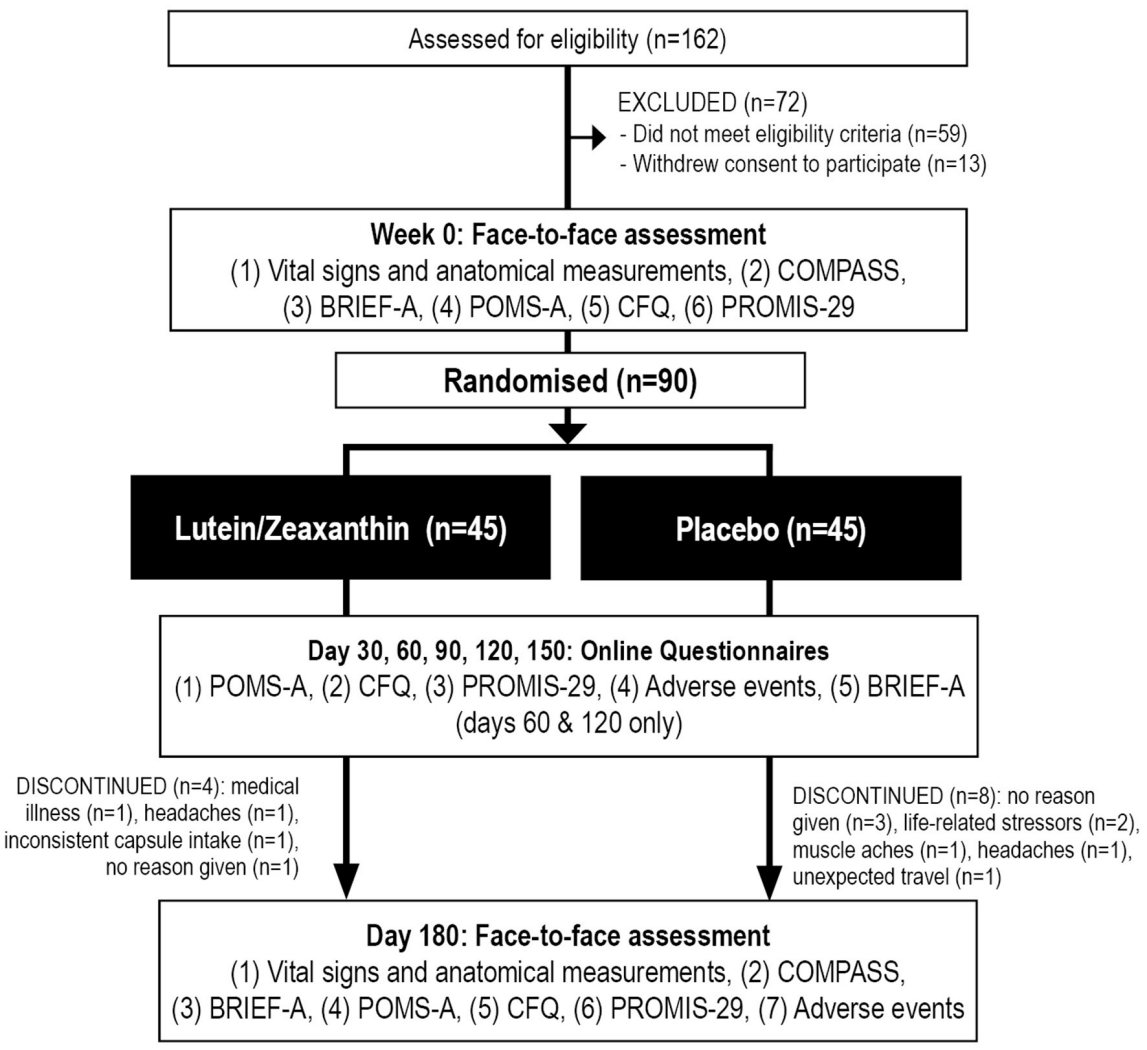
Systematic illustration of study design. BRIEF-A, Behavior Rating Inventory of Executive Function—Adult Version; CFQ, Cognitive Failures Questionnaire; COMPASS, Computerized Mental Performance Assessment System; POMS-A, Profile of Mood States, Abbreviated Version; PROMIS-29, Patient-Reported Outcomes Measurement Information System-29.

This was a two-arm, parallel-group, 180-day (6-month), single-center, randomized, double-blind, placebo-controlled trial (Figure 1). All participants gave informed consent, and the trial received ethics approval from the Human Research Ethics Committee at the National Institute of Integrative Medicine (approval number 0082E_2020). This study was prospectively registered with the Australian and New Zealand Clinical Trials Registry (Trial ID. ACTRN12621000038897).

### Sample Size Calculation

Based on a single outcome variable, an *a priori* power analysis was completed to estimate the required sample size. In a another trial examining the effects of lutein supplementation on episodic memory, an effect size of 0.6 was identified (25). Assuming a power of 80% and a type one error rate (alpha) of 5%, the number of participants required per group to find a treatment effect was estimated as 36. After allowing for an approximate 15% dropout rate, we aimed to recruit 45 participants per group.

### Recruitment and Randomization

Participants were recruited through social media and email databases between February and March 2021. Interested volunteers visited a website page that provided further information about the study and a link to complete a screening form that assessed for self-reported problems with memory and attention; history of psychiatric disorders or medical diseases; medication use; nicotine, alcohol, and other drug use; and the intake of vitamin and herbal supplements. The 4-item Patient Health Questionnaire (PHQ-4) was also completed to assess for symptoms of anxiety and depression (26). If judged as likely eligible, volunteers participated in a telephone interview where they were asked a series of questions to assess their eligibility and to obtain further demographic details. During this assessment, the Australian adaptation of the Modified Telephone Interview for Cognitive Status (TICS-M) was administered by a researcher (27). The TICS-M is validated against other cognitive screens such as the Mini-Mental State Examination (28). Suitable participants then completed a consent form (online) and attended an in-person assessment ~3–7 days after the interview. During the assessment, participants completed online versions of the Behavior Rating Inventory of Executive Function—Adult Version (BRIEF-A), Profile of Mood States, Abbreviated Version (POMS-A), Patient-Reported Outcomes Measurement Information System-29 (PROMIS-29), and Cognitive Failures Questionnaire (CFQ). Participants also completed several tasks from the Computerized Mental Performance Assessment System (COMPASS) (see Table 1).

**Table 1:** COMPASS tasks completed.

**Task**	**Score**
Word recall (immediate)	Number correct
Word recall (delayed)	Number correct
Location learning recall	Displacement score
Word recognition	Percentage correct
	Percentage correct
Picture recognition	Percentage correct
	Correct responses (milliseconds)
Numeric working memory	Percentage correct
	Correct responses (milliseconds)
	Percentage correct
Corsi blocks	Span score
Simple reaction time	Correct responses (milliseconds)
Choice reaction time	Correct responses (milliseconds)
Digit vigilance	Correct responses (milliseconds)
Stroop	Correct responses (milliseconds)

Consenting and eligible participants were randomly allocated to one of two groups (lutein/zeaxanthin or placebo). To ensure sequence concealment, a randomization calculator was used with the randomization structure consisting of 9 randomly permuted blocks, with 10 participants per block. Identification numbers were assigned to participants based on their order of enrolment in the study. All capsules were packaged in identical bottles labeled by two intervention codes (held by the sponsor until all data was collected). Study investigators and participants were blind to the treatment group allocation until all outcome data were collected.

### Participants

Inclusion criteria: male and female participants aged 40–75 years, with self-reported problems in memory or attention were recruited for this study as indicated by a positive response to the following question: Do you feel you have problems with your memory, attention, or concentration? Volunteers scored above the 5th percentile for their education, age, and sex on the Australian version of the Telephone Interview for Cognitive Status- modified version (TICS-M) and had a body mass index (BMI) between 18 and 35. Participants were fluent in English and consented (online) to all relevant aspects of the study.

Exclusion criteria: Ineligible participants were diagnosed with dementia (based on the revised National Institute on Aging-Alzheimer's Association criteria) and suffered from severe or unstable medical conditions such as cardiovascular disease; bleeding disorders; hypertension; type I diabetes; renal failure; hepatic disease; glaucoma; pulmonary disease; gastrointestinal disease; gallbladder disease; and neurodegenerative or neurological disease. Participants were also ineligible if they were diagnosed with a severe psychiatric disorder or scored >8 on the PHQ-4 (indicating moderate-to-severe anxiety and/or depression). A history of head injury (with a loss of consciousness), seizures, or stroke, any major surgeries over the last year, hearing loss that may impact the person's ability to complete the phone assessment, regular medication use including anti-coagulants, anticholinergics, acetylcholinesterase inhibitors, and steroids, and any medication change in the past 3 months or anticipation to change during the trial period were also exclusion criteria. Individuals taking vitamin or herbal supplements that may significantly affect study outcomes, a current or 12-month history of illicit drug abuse, and alcohol consumption of more than 14 standard drinks per week were also unable to participate in the trial.

### Interventions

Lutein/zeaxanthin and placebo capsules were matched for shape, color coating, and size. The active ingredient, supplied by Bio-gen Extracts Pvt Ltd, contained 10 mg of lutein and 2 mg of zeaxanthin in sunflower oil. These doses were chosen as they have been commonly used in previous randomized controlled trials (22, 23, 29). The placebo capsules comprised the same excipients as the active capsules (sunflower oil). All participants were instructed to take 1 capsule, in the evening, with or without food, for 180 days. Capsule compliance was assessed by asking participants to estimate the consistency of capsule intake (0–100%), recording the intake of evening capsule intake on a mobile phone pill reminder/ monitoring application, and by returning unused capsules at the final assessment. At the end of the study, treatment blinding was evaluated by asking participants to predict group allocation (placebo, lutein/zeaxanthin, or uncertain).

### Outcome Measures

#### Primary Outcome Measure

##### Computerized Mental Performance Assessment System (*COMPASS)*

The COMPASS is a computer application that presents cognitive tasks to assess memory and speed of performance, attention, and visual learning (Northumbria University, Newcastle upon Tyne, UK). Results on the COMPASS are sensitive to nutritional and dietary interventions (30, 31). The COMPASS was completed at baseline and day 180, and the cognitive tasks administered in this study are detailed in Table 1. Participants completed a brief practice run at each assessment to familiarize themselves with the cognitive tasks and then completed the battery of cognitive tasks as detailed in Table 1. To control for morning food and beverage intake, participants disclosed their breakfast intake during their baseline visit and were asked (and reminded the day before their final assessment) to eat the same breakfast on the morning of their final assessment. Participants were asked not to consume any alcohol on the evening before testing and not to consume any caffeinated beverage the morning of their assessment. All assessments were conducted between 8 and 11 am, with testing at baseline and day 180 occurring at approximately the same time for each participant.

#### Secondary Outcome Measures

##### Behavior Rating Inventory of Executive Function—Adult Version (*BRIEF-A)*

The BRIEF-A is a validated questionnaire of executive function in adults aged 18–90 years. The BRIEF-A contains 75 items within 9 nonoverlapping theoretically and empirically-derived clinical scales: Inhibit, Self-Monitor, Plan/Organize, Shift, Initiate, Task Monitor, Emotional Control, Working Memory, and Organization of Materials. These clinical scales combine for two index scores, the Behavioral regulation index, and the metacognition index (32). The BRIEF-A was completed at baseline, and days 60, 120, and 180.

##### Profile of Mood States, Abbreviated Version (*POMS-A)*

The POMS-A is a psychometrically-validated, 40-item self-report questionnaire that assesses a respondent's current mood state (33). Questions are rated on a 4-point scale (not at all to extremely), and scores are calculated for tension, anger, fatigue, depression, esteem-related affect, vigor, confusion, and total mood disturbance. The POMS-A was completed at baseline and days 30, 60, 90, 120, 150, and 180.

##### Patient-Reported Outcomes Measurement Information System-29 (*PROMIS-29)*

The PROMIS-29 is a validated self-report questionnaire that assesses the following seven domains: (1) Anxiety, (2) Depression, (3) Physical function, (4) Sleep disturbance, (5) Fatigue, (6) capacity to participate in social activities and roles, and (7) Pain intensity and interference (34). Higher scores on physical function and the ability to participate in social roles and activities imply better function, whereas lower scores in the other domains indicate an improvement in symptoms. The PROMIS-29 was completed at baseline and days 30, 60, 90, 120, 150, and 180.

##### Cognitive Failures Questionnaire (*CFQ)*

The CFQ is a 25-item self-report questionnaire that assesses the frequency of cognitive difficulties (35). The CFQ has sound psychometric properties (36), where lower scores indicate improved cognitive skills. The CFQ was completed at baseline and days 30, 60, 90, 120, 150, and 180.

##### Adverse Events

The tolerability of capsule intake was assessed every 30 days by an online question querying side effects that were believed to be due to capsule intake. Participants were also asked to contact researchers if they experienced any adverse effects.

### Statistical Analysis

For baseline data, an independent samples *t*-test was used to examine group data for continuous variables, and a Pearson's Chi-square test was used to examine categorical data. Five separate repeated-measures multivariate ANOVAs were conducted to examine change in scores on (1) computer-based measures of episodic memory (days 0 and 180), (2) computer-based measures of working memory (days 0 and 180), (3) computer-based measures of speed of performance (days 0 and 180), (4) self-report questionnaires (POMS-A total mood disturbance score, PROMIS-29 sub-scale scores, and the CFQ total score, days 0, 30, 60, 90, 120, 150, and 180), and (5) BRIEF-A index scores (Behavioral Regulation index and Metacognition index, days 60, 120, and 180). Episodic memory comprised scores on the following tasks: immediate word recall (number correct), delayed word recall (number correct), word recognition (percentage correct), picture recognition (percentage correct), numeric working memory (percentage correct), and location learning recall (displacement score). Working memory comprised scores on the following tasks: Corsi blocks (span score) and numeric working memory (percentage correct). Speed of information processing comprised the following measures (reaction time in milliseconds of correct responses): simple reaction time, choice reaction time, numeric working memory, picture recognition, word recognition, digit vigilance, and Stroop. These categorizations are consistent with other studies that have used the COMPASS as an outcome measure (37, 38). As a measure of visuospatial learning, a time x trial x group, repeated-measures ANOVA was conducted on the displacement scores (trials 1–6) on the location learning task. A visual inspection of Q-Q plots and analysis of skewness and kurtosis were used to assess the normality of residuals. This showed that self-report data were normally distributed. However, COMPASS scores were not normally distributed, so the data was winsorized whereby scores more than 3 standard deviations from the mean were substituted with the next highest value. Winsorizing is a robust approach to normalize data (39) and improved the normality of COMPASS data. To correct for violations of the sphericity assumption, where required, degrees of freedom were adjusted using the Greenhouse-Geisser approach. Participant data were included in the analyses of self-report outcomes if questionnaires were completed at day 30 [for missing values, last observation carried forward]. All results were analyzed using SPSS (version 26; IBM, Armonk, NY) using a critical *p*-value of ≤0.05 for all analyses. Because of the exploratory nature of this trial, the *p-*value was not adjusted for multiple testing. However, by using a step-down analysis, the type 1 error rate was minimized, whereby the multivariate ANOVA needed to be significant before proceeding to the exploration of univariate analyses.

## Results

### Study Population

#### Baseline Questionnaire and Demographic Information

As detailed in Figure 1, from 162 people who completed the online screening survey, 59 people did not meet the eligibility criteria, and 13 individuals withdrew consent to participate in the study. Ninety people participated in the study and 78 completed the study. Background details and baseline scores of the recruited sample are included in Tables 2, 3. Baseline demographics, questionnaire scores, and COMPASS test results were equivalent in the active and placebo groups, except for a slower reaction time in the numeric working memory task in participants in the placebo group. Eleven participants withdrew from the trial. Reasons for withdrawal included no reason given (*n* = 4), increased life stressors (*n* = 2), headaches (*n* = 2), worsening of an unrelated medical condition (*n* = 1), muscle aches (*n* = 1), and inconsistent capsule intake (*n* = 1).

**Table 2:** Baseline demographics details and questionnaire scores.

		**Placebo (*n =* 45)**	**Lutein/zeaxanthin (*n =* 45)**	***p*-value**
Age	Mean	60.04	58.78	0.480^a^
	SE	1.27	1.26	
Sex	Female (n)	35	36	0.796^b^
	Male (n)	10	9	
BMI	Mean	27.12	27.20	0.934^a^
	SE	0.59	0.67	
Systolic blood pressure (mmHg)	Mean	126.60	128.89	0.501^a^
	SE	2.29	2.50	
Diastolic blood pressure (mmHg)	Mean	82.58	82.89	0.866^a^
	SE	1.17	1.42	
Marital status	Single	18	11	0.114^b^
	Married/ defacto	27	34	
Educational level	Secondary	20	16	0.435^b^
	Tertiary	17	16	
	Post-graduate	8	13	
Exercise level (n)	Never/ rarely	8	7	0.519^b^
	1 to 2 times a week	5	10	
	3 to 5 times a week	15	15	
	6+ times a week	17	13	
Taking any prescription medication	Yes	21	26	0.291^b^
Taking nutraceutical/ phytoceutical	Yes	25	21	0.399^b^
TICS score	Mean	26.87	27.40	0.461^a^
	SE	0.56	0.46	
CFQ - Total score	Mean	43.16	43.33	0.946^a^
	SE	1.57	2.10	
BRIEF-A Behavioral Regulation	Mean	45.42	44.78	0.735^a^
	SE	1.21	1.46	
BRIEF-A Metacognition	Mean	66.84	65.87	0.728^a^
	SE	1.91	2.05	
PROMIS-29 Physical Function	Mean	18.98	19.18	0.593^a^
	SE	0.28	0.25	
PROMIS-29 Anxiety	Mean	7.11	7.40	0.756^a^
	SE	0.83	0.41	
PROMIS-29 Depression	Mean	6.07	6.44	0.503^a^
	SE	0.42	0.38	
PROMIS-29 Fatigue	Mean	10.40	10.02	0.648^a^
	SE	0.58	0.59	
PROMIS-29 Sleep disturbances	Mean	11.58	10.56	0.174^a^
	SE	0.60	0.45	
PROMIS-29 Ability to participate in social roles and activities	Mean	16.18	16.16	0.977^a^
	SE	0.57	0.54	
PROMIS-29 Pain interference	Mean	6.84	6.29	0.439^a^
	SE	0.556	0.45	
PROMIS-29 Pain intensity	Mean	2.38	1.84	0.203^a^
	SE	0.32	0.266	
POMS-A Total Mood Disturbance	Mean	90.69	91.02	0.914^a^
	SE	1.84	2.46	

a *Independent samples t-test*;

b *Chi-square analysis*.

**Table 3:** Baseline COMPASS scores.

		**Placebo (*n =* 45)**	**Lutein/zeaxanthin (*n =* 45)**	***p*-value^a^**
Immediate word recall (*n*)	Mean	5.18	5.47	0.469
	SE	0.28	0.28	
Delayed word recall (*n*)	Mean	3.07	3.18	0.804
	SE	0.30	0.33	
Simple reaction time (ms)	Mean	368.75	355.27	0.383
	SE	10.15	11.53	
Choice reaction time correct (%)	Mean	98.04	98.44	0.368
	SE	0.29	0.33	
Choice reaction time for correct responses (ms)	Mean	593.33	559.79	0.063
	SE	12.58	12.64	
Location learning trial 1 (displacement score)	Mean	16.58	14.91	0.330
	SE	1.36	1.02	
Location learning trial 2 (displacement score)	Mean	10.02	10.27	0.876
	SE	1.18	1.02	
Location learning trial 3 (displacement score)	Mean	7.27	5.47	0.195
	SE	1.13	0.80	
Location learning trial 4 (displacement score)	Mean	4.93	4.33	0.653
	SE	1.04	0.83	
Location learning trial 5 (displacement score)	Mean	2.60	2.64	0.955
	SE	0.58	0.54	
Location learning recall (displacement score)	Mean	4.18	3.73	0.637
	SE	0.84	0.63	
Numeric working memory correct (%)	Mean	93.85	94.67	0.480
	SE	0.92	0.68	
Numeric working memory reaction time for correct responses (ms)	Mean	1,213.00	1,098.80	0.039
	SE	39.26	37.78	
Word recognition correct (%)	Mean	76.89	76.59	0.886
	SE	1.41	1.49	
Word recognition reaction time for correct responses (ms)	Mean	1,399.35	1,357.50	0.639
	SE	57.15	68.06	
Picture recognition correct (%)	Mean	96.15	94.22	0.097
	SE	0.69	0.91	
Picture recognition reaction time for correct responses (ms)	Mean	1,036.03	1,001.78	0.326
	SE	27.26	21.39	
Corsi blocks span score	Mean	5.15	5.27	0.591
	SE	0.17	0.14	
Digit vigilance correct (%)	Mean	90.17	92.15	0.200
	SE	1.17	0.98	
Digit vigilance reaction time for correct responses (%)	Mean	471.44	469.23	0.767
	SE	5.80	4.63	
Stroop correct (%)	Mean	97.28	96.50	0.487
	SE	0.62	0.92	
Stroop reaction time for correct (ms)	Mean	1,313.24	1,291.83	0.754
	SE	47.04	49.15	

a *Independent samples t-test*.

### Outcome Measures

#### Primary Outcome Measure: COMPASS Scores

Changes in the cognitive tasks and cognitive categories across the two treatment conditions and ANOVA significance levels are detailed in Table 4. Repeated-measure MANOVAs revealed there was a statistically-significant between-group difference in change scores for episodic memory (*F*_5,72_ = 3.74, *p* = 0.005) but not working memory (*F*_2,75_ = 0.10, *p* = 0.903) or speed of performance (*F*_7,70_ = 0.44, *p* = 0.873). From baseline to day 180, episodic memory [immediate word recall (percentage correct), delayed word recall (percentage correct), location learning recall (displacement score), word recognition (percentage correct), and picture recognition (percentage correct) scores] increased significantly in the lutein/zeaxanthin group (*F*_5,36_ = 3.07, *p* = 0.021) but not the placebo group (*F*_5,32_ = 1.96, *p* = 0.112). Including baseline reaction time in numeric working memory as a covariate did not affect statistical outcomes.

**Table 4:** Change in COMPASS tasks.

		**Placebo (*n* = 37)**				**Lutein/Zeaxanthin (*n* = 41)**				**Univariate between-group *p*-value^b^**	**Multivariate between-group *p*-value^b^**
		**Day 0**	**Day 180**	**Change**	***p*-value^a^**	**Day 0**	**Day 180**	**Change**	***p*-value^a^**		
**Measures of episodic memory**											
Immediate word recall (*n*)	Mean	5.24	5.46	0.22	0.532	5.41	5.88	0.46	0.110	0.577	0.005
	SE	0.33	0.37	0.34		0.30	0.36	0.28			
Delayed word recall (*n*)	Mean	3.03	3.32	0.30	0.316	3.10	3.66	0.56	0.060	0.525	
	SE	0.34	0.36	0.29		0.34	0.38	0.29			
Location learning recall (displacement	Mean	4.24	5.89	1.65	0.115	3.51	1.49	−2.02	0.019	0.006	
score)	SE	0.90	0.92	1.02		0.66	0.71	0.83			
Word recognition correct (%)	Mean	77.66	76.49	−1.17	0.535	76.50	76.67	0.16	0.918	0.584	
	SE	1.56	1.85	1.87		1.54	1.54	1.57			
Picture recognition correct (%)	Mean	96.58	94.42	−2.16	0.045	94.80	97.15	2.36	0.006	0.001	
	SE	0.66	1.01	1.04		0.80	0.54	0.82			
**Measures of speed of performance**											
Simple reaction time (ms)	Mean	370.54	360.01	−10.53	0.405	355.13	347.50	−7.63	0.435	0.853	0.873
	SE	10.96	11.86	12.51		11.97	11.60	9.67			
Choice reaction time for correct	Mean	588.96	560.19	−28.78	0.024	563.86	554.10	−9.76	0.364	0.242	
responses (ms)	SE	13.70	10.72	12.23		13.30	12.71	10.62			
Numeric working memory reaction	Mean	1,178.86	1,126.99	−51.87	0.087	1,117.24	1,086.92	−30.32	0.349	0.624	
time for correct responses (ms)	SE	38.49	34.92	29.46		40.08	32.61	31.99			
Word recognition reaction time for	Mean	1,388.79	1,356.84	−31.95	0.662	1,309.05	1,306.40	−2.65	0.955	0.730	
correct responses (ms)	SE	57.28	62.93	72.56		59.80	59.34	46.71			
Picture recognition reaction time for	Mean	1,026.32	1,021.15	−5.17	0.843	1,003.87	1,061.95	58.07	0.119	0.170	
correct responses (ms)	SE	26.94	30.06	25.91		22.71	36.88	36.49			
Digit vigilance reaction time for	Mean	471.40	478.53	7.13	0.191	468.11	471.57	3.46	0.378	0.575	
correct responses (ms)	SE	6.53	6.30	5.35		4.45	3.98	3.88			
Stroop reaction time for correct	Mean	1,336.79	1,273.22	−63.58	0.218	1,310.36	1,270.80	−39.56	0.525	0.768	
responses (ms)	SE	54.45	45.31	50.74		52.36	43.16	61.77			
**Working memory**											
Corsi blocks (span score)	Mean	5.16	5.20	0.04	0.848	5.23	5.18	−0.05	0.760	0.729	0.903
	SE	0.18	0.19	0.19		0.15	0.16	0.16			
Numeric working memory	Mean	93.45	93.81	0.36	0.710	94.58	95.29	0.71	0.294	0.765	
(percentage correct)	SE	1.10	0.92	0.96		0.74	0.66	0.66			
**Other COMPASS tasks**											
Choice reaction time correct (%)	Mean	97.84	98.38	0.54	0.106	98.39	98.83	0.44	0.183	0.826	NA
	SE	0.34	0.28	0.33		0.36	0.25	0.32			
Digit vigilance correct (%)	Mean	90.45	92.55	2.10	0.127	93.01	92.74	−0.27	0.811	0.178	
	SE	1.27	1.21	1.35		0.89	1.13	1.13			
Stroop correct (%)	Mean	97.03	97.77	0.74	0.275	96.83	98.23	1.40	0.096	0.542	
	SE	0.74	0.46	0.67		0.92	0.53	0.82			

a *Repeated-measures ANOVA*;

b *Repeated-measures ANOVA, time × group interaction with medication and supplement use entered as covariates*.

An examination of individual COMPASS tasks revealed there were between-group differences in change scores for the correct responses in the picture recognition (*F*_1,76_ = 11.88, *p* = 0.001) and location learning recall (*F*_1,76_ = 7.86, *p* = 0.006) tasks. In the lutein/zeaxanthin group there was an improved performance in the picture recognition (*F*_1,40_ = 8.33, *p* = 0.006) and location learning recall tasks (*F*_1,40_ = 6.00, *p* = 0.019) from baseline to day 180.

**Figure 2:**
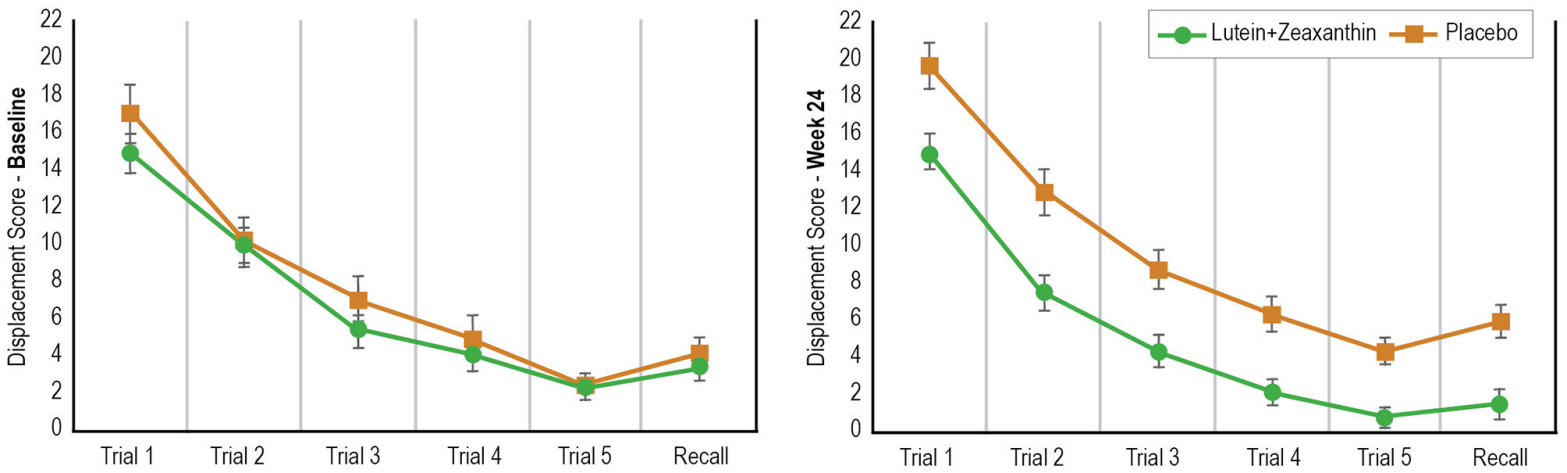
Change in Performance on the Location Learning Task from Baseline to Day 180.

In the location learning task (comprising 5 learning and one recall trial), a repeated-measures ANOVA time x group x trial analysis revealed a non-significant interaction (*F*_5,380_ =0.587, *p* = 0.710) (Table 5). However, there was a statistically-significant time x group interaction indicating an overall better performance on the location learning task in the lutein/zeaxanthin group compared to the placebo group (*F*_1,76_ = 11.60, *p* = 0.001) (Figure 2).

**Table 5:** Change in location learning scores.

		**Placebo (*n* = 37)**		**Lutein/zeaxanthin (*n* = 41)**		**Time × group interaction**	**Time × trial × group interaction**
Location learning trial 1	Mean	17.08	19.57	14.98	15.05	0.001	0.710
(displacement score)	SE	1.53	1.20	1.10	0.98		
Location learning trial 2	Mean	10.32	12.84	9.90	7.37		
(displacement score)	SE	1.28	1.21	1.05	0.94		
Location learning trial 3	Mean	7.14	8.73	5.44	4.41		
(displacement score)	SE	1.19	1.02	0.86	0.84		
Location learning trial 4	Mean	5.14	6.35	4.27	2.07		
(displacement score)	SE	1.14	0.89	0.87	0.67		
Location learning trial 5	Mean	2.62	4.35	2.32	0.71		
(displacement score)	SE	0.59	0.67	0.53	0.38		
Location learning recall	Mean	4.24	5.89	3.51	1.49		
(displacement score)	SE	0.90	0.92	0.66	0.71		

*A decrease in score indicates an improvement in performance*.

#### Secondary Outcome Measures: Self-Report Questionnaires

Changes in scores on the self-report questionnaires across the two conditions and repeated measures ANOVA significance levels are detailed in Table 6. A multivariate analysis showed there was no statistically-significant time x group interaction comprising the POMS-A total mood disturbances score, PROMIS-29 sub-scale scores, and the CFQ total score (*p* = 0.301); or the BRIEF-A index scores (*p* = 0.771). There were statistically-significant reductions in the total CFQ score and the BRIEF-A index scores (behavioral regulation and metacognition index) in both the lutein/zeaxanthin and placebo groups.

**Table 6:** Change-in-self-report-questionnaires.

		**Placebo (*n* = 45)**									**Lutein/zeaxanthin (*n* = 45)**									**Univariate between-group *p*-value^b^**	**Multivariate between-group *p*-value^b^**
		**Day 0**	**Day 30**	**Day 60**	**Day 90**	**Day 120**	**Day 150**	**Day 180**	**Change**	***p*-value^a^**	**Day 0**	**Day 30**	**Day 60**	**Day 90**	**Day 120**	**Day 150**	**Day 180**	**Change**	***p*-value^a^**		
POMS-A Total	Mean	90.69	94.07	91.49	93.11	90.87	89.47	90.91	0.22	0.421	91.02	92.16	94.38	91.24	92.13	93.64	88.51	−2.51	0.139	0.210	0.301
Mood Disturbance	SE	1.84	2.30	2.19	2.42	2.37	2.18	3.02	2.87		2.46	2.77	2.88	2.82	2.93	2.70	2.43	2.43			
PROMIS-29	Mean	18.98	19.00	19.07	18.71	19.07	19.22	19.09	0.11	0.798	19.18	18.91	18.82	18.36	18.67	18.76	18.67	−0.51	0.407	0.810	
Physical function	SE	0.28	0.22	0.28	0.48	0.24	0.21	0.26	0.22		0.25	0.31	0.34	0.54	0.43	0.35	0.36	0.26			
PROMIS-29	Mean	7.11	6.00	5.93	6.36	6.09	5.82	6.36	−0.76	0.090	7.40	6.60	6.93	6.20	6.51	6.42	6.62	−0.78	0.014	0.561	
Anxiety	SE	0.83	0.35	0.34	0.36	0.36	0.32	0.35	0.74		0.41	0.42	0.47	0.37	0.42	0.40	0.38	0.36			
PROMIS-29	Mean	6.07	5.49	5.38	5.84	5.62	5.67	5.51	−0.56	0.314	6.44	5.89	6.24	5.89	6.31	6.27	6.09	−0.36	0.613	0.693	
Depression	SE	0.42	0.34	0.29	0.39	0.34	0.32	0.32	0.35		0.38	0.38	0.41	0.37	0.44	0.43	0.41	0.39			
PROMIS-29	Mean	10.40	9.27	8.82	8.96	8.91	8.60	9.36	−1.04	0.001	10.02	9.04	9.49	9.53	8.76	9.38	9.24	−0.78	0.292	0.388	
Fatigue	SE	0.58	0.53	0.58	0.53	0.48	0.45	0.49	0.50		0.59	0.49	0.56	0.50	0.50	0.58	0.54	0.55			
PROMIS-29	Mean	11.58	11.00	10.87	10.76	10.11	10.64	10.67	−0.91	0.131	10.56	9.96	10.62	10.16	9.62	9.87	9.78	−0.78	0.145	0.869	
Sleep disturbance	SE	0.60	0.57	0.60	0.59	0.54	0.53	0.52	0.63		0.45	0.46	0.50	0.50	0.51	0.54	0.53	0.48			
PROMIS-29 Social	Mean	16.18	16.40	16.87	16.78	16.62	16.76	16.82	0.64	0.647	16.16	16.24	16.09	16.09	16.47	16.38	16.18	0.02	0.985	0.866	
roles and activities	SE	0.57	0.53	0.53	0.52	0.53	0.50	0.56	0.45		0.54	0.57	0.59	0.57	0.59	0.59	0.55	0.56			
PROMIS-29 Pain	Mean	6.84	7.18	6.73	6.73	6.84	6.76	7.04	0.20	0.916	6.29	6.40	6.76	6.40	6.04	6.44	6.73	0.44	0.719	0.838	
interference	SE	0.56	0.63	0.54	0.57	0.58	0.62	0.65	0.42		0.45	0.52	0.46	0.53	0.44	0.53	0.56	0.59			
PROMIS-29 Pain	Mean	2.38	2.76	2.58	2.84	2.56	2.58	2.58	0.20	0.745	1.84	2.69	2.73	2.33	2.13	2.47	2.64	0.80	0.011	0.399	
intensity	SE	0.32	0.38	0.34	0.37	0.37	0.37	0.37	0.31		0.27	0.31	0.32	0.35	0.32	0.36	0.37	0.32			
CFQ	Mean	43.16	37.02	33.40	32.91	33.13	31.67	34.24	−8.91	<0.001	43.33	37.51	35.62	33.82	33.11	33.33	34.58	−8.76	<0.001	0.759	
	SE	1.57	1.44	1.44	1.54	1.52	1.62	1.53	1.46		2.10	2.06	1.86	1.85	1.94	1.88	2.07	1.41			
BRIEF-A Behavioral	Mean	45.42		42.00		41.91		42.82	−2.60	<0.001	44.78		42.16		42.87		42.98	−1.80	0.002	0.554	0.771
regulation index	SE	1.21		1.44		1.39		1.56	0.93		1.46		1.41		1.53		1.40	0.74			
BRIEF-A	Mean	66.84		62.29		61.84		63.36	−3.49	<0.001	65.87		60.87		61.71		62.73	−3.13	<0.001	0.839	
Metacognition index	SE	1.91		1.80		2.03		2.01	1.28		2.05		1.97		2.13		2.08	1.14			

a *Repeated-measures ANOVA*;

b *Repeated-measures ANOVA, time x group interaction*.

#### Intake of Supplements

Bottles with remaining capsules were returned on the day 180 assessment, and a daily medication monitoring phone application was completed by participants. Based on these details, 96% of participants who completed the study took >80% of their capsules.

#### Efficacy of Participant Blinding

To assess the effectiveness of condition concealment, at the end of the study, participants were asked to predict group allocation (i.e., placebo, lutein/zeaxanthin, or unsure). Group concealment was satisfactory as 80% of participants were either unsure or incorrectly predicted group allocation.

#### Adverse Events

The incidence of self-reported adverse events is detailed in Table 7. There was a trend for more self-reported adverse events in the placebo group (*n* = 12) compared to the lutein/zeaxanthin group (*n* = 8). No serious adverse events were reported by participants, although two participants withdrew due to reported ongoing headaches (one in the placebo and one in the lutein/zeaxanthin group), and one person withdrew due to self-reported muscle pain (placebo group). There were no reports of any adverse events in 77% of participants. There were no statistically-significant between-group differences in changes in BMI (*p* = 0.615), systolic (*p* = 0.318), or diastolic (*p* = 0.849) blood pressure over time.

**Table 7:** Frequency of self-reported adverse events.

	**Placebo**	**Lutein/zeaxanthin**
Digestive disturbances	3	3
Headache	1	3
Sleep disturbances	2	1
Muscle pain	2	
Nausea	1	
Weight gain	1	
Skin rash		1
Dizziness	1	
Dry mouth	1	
Total number of adverse effects	12	8

## Discussion

In this 6-month, randomized, double-blind, placebo-controlled trial, supplementation with lutein and zeaxanthin at a daily dose of 10 and 2 mg, respectively, was associated with greater improvements in visual episodic memory compared to the placebo. Superior improvements in visual learning, as measured by the computerized location learning task, were also observed. However, there were no other between-group differences in changes in other computer-based cognitive tasks and self-report measures of executive function, memory, mood, or physical function. Lutein and zeaxanthin supplementation was well-tolerated with no reports of significant adverse effects.

The effects of lutein and zeaxanthin on cognitive performance have been investigated in several human trials. Consistent with the findings from this study, improvements in visual memory and performance have identified in several studies. In a placebo-controlled study on young adults aged 18–30 years, 12 months of lutein and zeaxanthin supplementation administered at the equivalent dose used in this study was associated with improvements in an immediate and 30-min delayed visual memory task comprising the presentation of shapes and symbols (23). In another 12-month, placebo-controlled trial on adults aged 18 years and older with a mean age of 45 years, supplementation with lutein, zeaxanthin, and *meso*-zeaxanthin improved performance on visual episodic memory as measured by a paired-associated learning task (25). Improvements in verbal recognition memory were also observed. Consistent with the results from these studies, improvements in visual episodic memory as measured by a 30-min delayed picture recognition task and location learning task were observed in this study. Further confirmation of improvements in visual cognitive performance is provided by superior performance on a computerized location learning task. Compared to the placebo, participants in the lutein and zeaxanthin group consistently performed better at each trial. The results from this trial suggest that supplementation with lutein and zeaxanthin may improve visual memory and learning in people aged 40–75 years (average age of 59 years), and when delivered over a shorter treatment duration (6 months) compared to previous trials (12 months). However, the findings from this study do contrast with the findings from a 12-month study conducted on community-dwelling older adults where supplementation was associated with increases in complex attention, cognitive flexibility and executive function, but not visual memory (22). Differences in the age of the populations recruited (mean age of 74 years vs. 59 years in this study) and length of supplementation (12 months vs. 6 months in this study) may account for the discrepancy in these findings.

The effects of lutein and zeaxanthin on visual memory and performance may have important implications for the prevention of cognitive decline as a relationship between visual memory and cognitive decline has been identified. In a 2-year longitudinal study, better visual memory was associated with a lower risk of cognitive deterioration up to 2 years later (40). In another study, a poorer spatial delayed recall was associated with more rapid conversion from pre-MCI to MCI. Specifically, pre-MCI older-age individuals with superior delayed spatial memory had a 3.8 times higher probability of stabilized cognitive performance compared to individuals with inferior spatial memory (41). Moreover, in a longitudinal study, poorer visual memory performance was associated with an increased risk of Alzheimer's disease up to 15 years later (42). The location learning task, a measure of visuospatial learning and recall, is also impaired in adults with dementia, older adults, and stroke sufferers (43, 44).

How lutein and zeaxanthin may improve memory requires further investigation and was not examined in this study. Because episodic memory is believed to be affected by neural components in the cortex (parahippocampal cortex, perirhinal cortex, and the entorhinal cortex), cortical and subcortical structures, and circuits within the hippocampus and medial temporal lobe, it is possible that lutein and zeaxanthin strengthened memory processes in these brain regions (45). Visual memory, in particular, is associated with activation of both anterior and posterior temporal cortices. Posterior temporal cortical regions seem to be involved in the retrieval of category-specific aspects of visual memory, while anterior areas of the temporal cortex are involved with category-independent visual memory (46). Moreover, deficits in spatial memory can occur after damage to the hippocampus (47) or parietal cortex lesions (48, 49), making these brain regions other potential areas targeted by lutein and zeaxanthin. Lutein and zeaxanthin may provide neuroprotection in these brain regions due to their antioxidant and anti-inflammatory properties (11, 50). Animal studies have also indicated lutein and zeaxanthin may reduce neurodegeneration by improving neurotrophic factors and synaptic proteins, and oxidative capacity in the cerebral cortex (51). In a human trial, 6 months of daily supplementation with macular xanthophylls (lutein, zeaxanthin and the zeaxanthin isomer meso-zeaxanthin) in healthy, young adults reduced serum interleukin-1β, and increased serum antioxidant capacity and brain-derived neurotrophic factor (52).

## Study Limitations and Directions for Future Research

Even though the results from this study suggest that supplementation with lutein and zeaxanthin is associated with improvements in visual memory and learning, several study limitations influence the robustness and generalizability of the findings. As participants were assessed on two occasions, 6-months apart, a portion of the improvements in cognitive performance may be associated with practice effects. However, because greater visual memory and learning improvements were observed in the active treatment group, lutein and zeaxanthin seem to have additional influences in these areas. Visual memory and learning were assessed by a 30-min delayed picture recognition task, a computerized location learning task (5 trials), and a 30-min delayed computerized location learning recall task. An examination into the effects of lutein and zeaxanthin supplementation over varying recall periods will be important to understand the sustainability of these ingredients on visual performance. Cognitive tests were also assessed using computer-based tasks where participants were required to complete tasks in a non-distracting environment. The real-world implications of improvements in cognitive performance, therefore, require further investigation. Moreover, despite improvements identified in visual memory computer-based tasks, results from self-report measures of memory and executive function indicated no between-group differences over time. In future trials, a more comprehensive battery of visual memory tasks, accompanied by objective tests such as measures in neural activity including functional magnetic resonance imaging, electroencephalogram activity, and neuroimaging, will help clarify the effects of lutein and zeaxanthin on the brain during a visual task. In a study by Mewborn et al. (53), 12 months of lutein and zeaxanthin supplementation in older-age adults had no significant effect on changes in global brain volume including global gray matter, global white matter, and white matter hypo-intensity. Because changes in MPOD provide a measure of lutein and zeaxanthin concentration in the brain (8, 19), examining the relationship between changes in MPOD and visual memory over time will also be helpful. Moreover, it will be important to clarify whether people with reduced MPOD experience greater cognitive benefits from lutein and zeaxanthin supplementation. Measurements of changes in blood concentrations of lutein and zeaxanthin, and monitoring of the dietary intake of lutein and zeaxanthin will also be important. This is especially pertinent as conducting studies on nutrients that are contained in everyday foods can be challenging and ideally should be adequately monitored or controlled for in clinical trials. In future studies, it will be helpful to understand the potential mechanisms associated with lutein and zeaxanthin supplementation. An investigation into changes in markers of inflammation, oxidative stress, and neurotrophins such as brain-derived neurotrophic factor, and their relationship with changes in cognitive performance will be important. In this study, healthy, community-dwelling adults aged 40 to 75 years, with self-reported memory complaints were recruited. The effects of lutein and zeaxanthin supplementation in people diagnosed with MCI or neurodegenerative diseases such as Alzheimer's disease requires further investigation. The effects of supplementation on cognitive function across diverse age groups, as a preventative or treatment for cognitive impairment, for more extended treatment periods, and at different doses will also help to further clarify the benefits of lutein and zeaxanthin supplementation. Lutein and zeaxanthin administration has also been associated with improvements in visual function, which could account for the changes in visual memory observed in this study. Assessing changes in visual function over time and their relationship to changes in visual memory will be helpful.

In summary, the results from this study suggest that supplementation with lutein and zeaxanthin for 6 months in community-dwelling adults with self-reported cognitive complaints is associated with improvements in visual memory and learning. However, the treatment had no other effect on other computer-based measures of cognitive performance or self-report measures of cognition, memory, mood, or physical function. Further trials will be essential to clarify the potential benefits of lutein and zeaxanthin supplementation in diverse populations over varying intervention periods, and utilizing additional measures of change in cognitive performance and neurological activity over time.

## Data Availability Statement

The raw data supporting the conclusions of this article will be made available by the authors, without undue reservation.

## Ethics Statement

The studies involving human participants were reviewed and approved by National Institute of Integrative Medicine. The patients/participants provided their written informed consent to participate in this study.

## Author Contributions

AL and SS designed the research study and conducted the research. AL, PD, and SS wrote the paper. All authors read and approved the final manuscript.

## Funding

This study received funding from Bio-gen Extracts Pvt., Ltd. The funder was not involved in data collection, analysis, interpretation of data, writing of the manuscript, or the decision to submit it for publication.

## Conflict of Interest

AL is the managing director of Clinical Research Australia, a contract research organization that has received research funding from nutraceutical companies. AL has also received presentation honoraria from nutraceutical companies. SS is an employee of Clinical Research Australia. The remaining author declares that the research was conducted in the absence of any commercial or financial relationships that could be construed as a potential conflict of interest.

## Publisher's Note

All claims expressed in this article are solely those of the authors and do not necessarily represent those of their affiliated organizations, or those of the publisher, the editors and the reviewers. Any product that may be evaluated in this article, or claim that may be made by its manufacturer, is not guaranteed or endorsed by the publisher.
